# Role of the Medial Orbitofrontal Cortex and Ventral Tegmental Area in Effort-Related Responding

**DOI:** 10.1093/texcom/tgaa086

**Published:** 2020-11-26

**Authors:** Alexandra Münster, Angeline Votteler, Susanne Sommer, Wolfgang Hauber

**Affiliations:** 1 Systems Neurobiology Research Unit, University of Stuttgart, Stuttgart D-70569, Germany; 2 Department of Neurobiology, University of Stuttgart, Stuttgart D-70569, Germany

**Keywords:** disconnection, effort, GABA, orbitofrontal cortex, ventral tegmental area

## Abstract

The posterior subdivision of the medial orbitofrontal cortex (mOFC-p) mediates the willingness to expend effort to reach a selected goal. However, the neural circuitry through which the mOFC-p modulates effort-related function is as yet unknown. The mOFC-p projects prominently to the posterior ventral tegmental area (pVTA). Therefore, we analyzed the role of the mOFC-p and interactions with the pVTA in effort-related responding using a combination of behavioral, pharmacological, and neural circuit analysis methods in rats. Pharmacological inhibition of the mOFC-p was found to increase lever pressing for food under a progressive ratio (PR) schedule of reinforcement. These findings provide further support for a modulation of effort-related function by the mOFC-p. Then, we investigated effects of disconnecting the mOFC-p and pVTA on PR responding using unilateral pharmacological inhibition of both areas. This asymmetric intervention was also found to increase PR responding suggesting that the mOFC-p controls effort-related function through interactions with the pVTA. Possibly, a reduced excitatory mOFC-p drive on pVTA gamma-aminobutyric acid (GABA)ergic relays disinhibits VTA dopamine neurons which are known to support PR responding. Collectively, our findings suggest that the mOFC-p and pVTA are key components of a neural circuit mediating the willingness to expend effort to reach a goal.

## Introduction

The orbitofrontal cortex (OFC) is an anatomically and functionally heterogeneous region that supports learning and decision-making in multiple ways, for instance, by providing predictions about outcomes associated with actions (Rudebeck and Murray 2014; Wilson et al. 2014). The medial subregion of the OFC has been specifically linked to motivational states and control of goal-directed action (Izquierdo 2017). Our recent findings suggest that the posterior subdivision of the medial OFC (mOFC-p) supports functions related to response effort. For instance, pharmacological stimulation and inhibition of the mOFC-p bidirectionally altered instrumental responding of rats in a progressive ratio (PR) task that demands increasingly more effort for a fixed outcome (Münster and Hauber 2018). However, to date, the neural circuitry through which the mOFC-p modulates effort-related responding is unknown.

Anatomical studies revealed major projections from the mOFC-p to the posterior ventral tegmental area (pVTA) (Gabbott et al. 2005; Hoover and Vertes 2011). Moreover, electrophysiological recordings indicated that the OFC controls VTA firing. For instance, an electrical stimulation of the OFC inhibited VTA dopamine neurons (Lodge 2011; Takahashi et al. 2011). Given that the output of the OFC is excitatory (Geisler et al. 2007), OFC stimulation was supposed to reduce VTA dopamine neuron activity via inhibitory relays such as VTA gamma-aminobutyric acid (GABA) ergic interneurons (Takahashi et al. 2011). In line with this account, microinjection of GABA_A/B_ receptor agonists into the pVTA induced stimulant behavioral effects and, for instance, enhanced motor activity (Arnt and Scheel-Krueger 1979; Wirtshafter and Klitenick 1989; Boehm 2nd et al. 2002). Stimulant behavioral effects were accounted for by an inhibition of pVTA GABA interneurons resulting, in turn, in an increased dopamine neuron activity (Kalivas et al. 1990; Kalivas 1993; Boehm 2nd et al. 2002). In view of the key role of VTA dopamine neurons in effort-related function (Boekhoudt et al. 2018), such a mechanism would also explain our observation that inhibition of the mOFC-p enhanced effort-related responding (Münster and Hauber 2018). Conceivably, a reduced excitatory drive on pVTA GABAergic interneurons could disinhibit pVTA dopamine neurons and enhance effort-related responding. Given these data, we hypothesized that the mOFC-p governs effort-related responding via GABAergic mechanisms in the pVTA.

Using a combination of behavioral, pharmacological, and neural circuit analysis methods, we examined in the current study how manipulations of the mOFC-p and its interactions with the pVTA altered PR responding. Unlike in our previous study (Münster and Hauber 2018), a novel PR task variant was used here in all experiments. This task variant does not involve a breakpoint (defined as the ratio at which an animal stops responding), a motivational index with inherent limitations (Stewart 1975; Bradshaw and Killeen 2012). We first analyzed the effects of mOFC-p inhibition on PR responding using local infusion of the GABA_A/B_ receptor agonists muscimol/baclofen (M/B) (Experiment 1). Then, we investigated the effects of a disconnection of mOFC-p and pVTA on PR responding by means of unilateral intra-mOFC-p M/B infusion in combination with contralateral intra-pVTA M/B infusion (Experiment 2). A unilateral intra-mOFC-p M/B infusion was presumed to inhibit pVTA GABA interneurons and to disinhibit pVTA dopamine neurons selectively within the same hemisphere because mOFC–VTA projections are largely uncrossed (Bradfield et al. 2018). Likewise, a contralateral intra-pVTA M/B infusion was presumed to inhibit pVTA GABA interneurons and to disinhibit pVTA dopamine neurons within the contralateral hemisphere. This asymmetric (“crossed”) intervention is thought to enhance pVTA dopamine neuron activity in both hemispheres thereby invigorating PR responding. In a subsequent experiment, we used asymmetric microinfusion techniques to further investigate the interactions of mOFC-p and pVTA GABAergic mechanisms (Experiment 3). We tested whether unilateral M/B infusion into the mOFC-p in combination with a contralateral infusion of GABA_A_ receptor antagonist picrotoxin into the pVTA altered PR responding. As pVTA microinjections of picrotoxin, unlike muscimol, did not modify motor activity and supposedly pVTA dopamine neuron activity (Arnt and Scheel-Krueger 1979), this asymmetrical intervention should not disinhibit pVTA dopamine neuron activity in both hemispheres and, therefore, should not invigorate PR responding. Experiment 3 may thus help to verify whether the mOFC drives PR responding by inhibition rather than stimulation of GABAergic mechanisms in the pVTA.

## Materials and Methods

All animal experiments were conducted according to the German Law of Animal Protection and were approved by the proper authorities.

### Subjects and Apparatus

Male Sprague Dawley rats (Janvier) were employed in all experiments, each using a within-subjects design, and were tested in standard operant conditioning chambers for PR responding. Rats were housed in groups in transparent plastic cages (55 × 39 × 27 cm; Tecniplast) in a temperature- and humidity-controlled room (20 ± 2 °C, 50–60%) on a 12:12 h light:dark cycle (lights on at 06:00 h). Throughout the experiments, they had ad libitum access to water. Standard laboratory maintenance chow (Altromin) was given ad libitum for 10 days after arrival; thereafter, food was restricted to 15 g per animal per day to maintain them on approximately 85% of their free-feeding weight. Training and testing took place in identical operant conditioning chambers (24 × 21 × 30 cm; Med Associates) housed within sound-attenuating cubicles. Each chamber was equipped with a pellet dispenser positioned in the middle of the wall that delivered a 45-mg pellet as reinforcer (termed here simply as “reward”). Each chamber also contained two retractable levers located on either side of the receptacle. A 24 V/3 W house light mounted on the top center of the opposite wall illuminated the chambers. A computer system (MedPC Software; Med Associates) controlled the equipment and recorded the data.

### Progressive Ratio Task

In our previous study (Münster and Hauber 2018), we used a PR task variant with a session duration of 60 min that involved a breakpoint defined as the highest completed ratio after which an animal stops responding. In the current study, a PR task variant was used, which included a moderate and linear schedule of ratio increases and demanded increasingly higher response rates but prevented the animals to completely stop lever pressing during the session. Moreover, to reduce confounding effects of motor fatigue and satiety on RP responding toward the end of long PR sessions, session time was constrained to 16 min. In the first minute, each lever press resulted in a reward delivery. Thereafter, the required lever presses increased with every minute until the final ratio of 140 (1–5–10–20–30–…–140). If a ratio was not completed, conducted lever presses were assigned to the following ratio. Our pilot experiments revealed that this PR task variant is sufficiently sensitive to bidirectional changes in PR responding following mOFC-p manipulations. In particular, it is sensitive to facilitated PR responding in rats with mOFC-p inactivation already shown in earlier studies (Münster and Hauber 2018) and thus avoids ceiling effects.

### Data Evaluation and Statistics

For each session, cumulative lever presses as well as running lever presses were evaluated as well as the mean postreinforcement pause across all trials, that is, the break after reward intake until the animal resumed lever pressing and the mean magazine latencies across all trials, that is, the latencies from reward delivery to magazine entry. Cumulated lever presses provide an overview on within-session lever pressing across increasing FR-values, and running lever presses (lever presses per minute) allow for a more detailed analysis of treatment effects on response rate shifts within a session. In addition, postreinforcement pauses and magazine latencies were assessed to check for treatment effects on motor capacity.

Within-subject designs were used for each experiment. Data were given as means ± standard error of the mean (SEM) throughout the paper. Data were subjected to repeated-measures analysis of variance (ANOVA). In addition, a paired *t*-test was used or if appropriate, a nonparametric test, that is, the Wilcoxon paired rank test. In addition, the effect size *r* for treatment effects on cumulative lever presses was calculated from the final FR-value from each experiment. Calculation was based on the *F*-value of a planned comparison of lever presses across treatments on the final FR-value using the following equation (Fields 2013):

$$r=\sqrt{\frac{F\left(1,{df}_R\right)}{F\left(1,{df}_R\right)+{df}_R}}.$$

All statistical computations were performed with STATISTICA (version 7.1, StatSoft). The level of statistical significance (α-level) was set at *P* ≤ 0.05.

### Experiment 1

Here we examined the effects of intra-mOFC-p microinfusions of the GABA_A/B_ agonists M/B on PR responding in rats (initial sample size, *N* = 20) weighing 370–480 g at the onset of testing. Rats included in Experiment 1 were implanted with guide cannulae aimed at the mOFC-p and pVTA. Note that these rats were also used in Experiment 2. In Supplementary experiments, we explored the effects of optogenetic mOFC-p stimulation on PR responding (Experiment S1) and c-Fos expression in the mOFC-p (Experiment S2) (see supplementary materials).

#### Behavioral Procedure

First, all animals received one magazine training session in which casein pellets (45-mg dustless precision pellets; Bioserv) were delivered on an independent random-time schedule (RT-60) with the lever withdrawn. The session lasted until 30 rewards were given. After magazine training, rats were given three sessions on a continuous reinforcement (CRF) schedule where each lever press resulted in the delivery of one casein pellet. Each session lasted for 30 min or until 100 rewards were given, whichever came first. Next, rats were trained in a PR task in which the required amount of lever presses to obtain a reward increased over time. In the first minute, each lever press resulted in a reward delivery. Thereafter, the required lever presses increased with every minute until the final FR 140 (1–5–10–20–30–…–140). Each session lasted for 16 min. Subsequently, animals were implanted bilaterally with cannulae aimed at the mOFC-p and the pVTA. Rats were retrained in the PR task for 3 additional days prior to the first microinfusion. Between test days, animals were given 3 days of PR task training. Drug effects were assessed in the PR task using a within-subjects design. That is, in the first test, half of the animals received intra-mOFC-p M/B, and the other half received intra-mOFC-p saline microinfusions. The reverse assignment was used in the second PR test. After completion of behavioral testing, animals were euthanized to control for cannula placements.

#### Surgery

For stereotaxic surgery, rats were anesthetized with a mixture of ketamine (90 mg/kg i.p.; Medistar GmbH) and xylazine (10 mg/kg Rompun i.p.; Bayer AG) and were secured in a stereotaxic apparatus with atraumatic ear bars (David Kopf Instruments). Bilateral stainless steel guide cannulae (0.8 mm outer diameter) aimed at the posterior mOFC-p as well as unilateral guide cannulae aimed at the pVTA were implanted using standard stereotaxic procedures (Bohn et al. 2003). The coordinates for the mOFC-p with reference to the atlas of Paxinos and Watson (1997) were: AP = +4.4 mm; ML = ±2.5 mm; DV = −4.5 mm, with the guide cannula positioned in an angle of 25° from the midline (tooth bar 3.3 mm below the interaural line). Coordinates were chosen to target the mOFC-p as detected by Bradfield et al. (2018). The coordinates for the pVTA were: AP = −6.0 mm; ML = ±2.1 mm; DV = −8.0 mm, relative to bregma with the guide cannula positioned in an angle of 10° (pVTA) from the midline. Coordinates were selected to target the pVTA based on data by Sanchez-Catalun et al. (2014) which showed that the borderline between anterior and pVTA was about AP: −5.5 mm. The guide cannulas were occluded by stainless steel stylets. Rats were allowed to recover for at least 3 days.

#### Microinfusion Procedure

To adapt rats to the microinjection procedure, they received sham microinjections including handling, insertion of microinjection cannulas dummies, and operation of the microinjection pump (without running a microinjection) on the last training day. Infusions were made via 30-gage microinjection cannulae at a rate of 0.3 μL/min by a microsyringe pump. For mOFC-p inactivation, a solution containing the GABA_B_ agonist baclofen (Sigma-Aldrich) and the GABA_A_ agonist muscimol (Sigma-Aldrich) was infused bilaterally into the mOFC-p. All drugs were dissolved in physiological saline at a final concentration of 125 ng/0.3 μL. Bilateral microinjections of 0.3 μL saline served as the control. Microinjection cannulae were left in position for an additional 1 min to allow for diffusion. Thereafter, each rat was placed in its home cage for 10 min before behavioral testing.

#### Histology

After completion of the behavioral testing, rats were euthanized and perfused transcardially with 0.01% heparin sodium salt in PBS, followed by 4% paraformaldehyde in PBS. Brains were removed, fixed in 4% formalin for 24 h, and stored in 30% glucose. Brains were frozen and coronal brain sections (35 μm) were collected, mounted on coated slides, and stained with cresyl violet. Cannula placements of rats were verified with reference to the atlas of Paxinos and Watson (1997). Two rats died during surgery, two other rats were excluded due to guide cannulae occlusion that emerged during Experiment 1, one rat was excluded because of incorrect mOFC-p cannula placement, and another rat was excluded because it failed to respond to the lever and did not receive any reward during PR testing. Hence, the final sample size in Experiment 1 was *N* = 14.

### Experiment 2

Here, we analyzed the effects of a disconnection of mOFC-p and pVTA on PR responding using rats (initial sample size, *N* = 20) also used in Experiment 1.

#### Behavioral Procedures and Microinfusions

Subjects were implanted with bilateral mOFC-p guide cannulae and unilateral VTA guide cannulae (for microinfusion procedures and surgery, see Experiment 1; note that Experiment 2 was performed prior to Experiment 1).

Rats were trained and tested for PR responding as described in Experiment 1. Each rat received three microinfusions on separate PR test days in a pseudorandom order: (1) unilateral mOFC-p microinfusion of M/B combined with a contralateral pVTA microinfusions of M/B (disconnection), (2) unilateral mOFC-p microinfusion of M/B combined with an ipsilateral pVTA microinfusion of M/B (disconnection control), and (3) unilateral mOFC-p microinfusion of saline combined with a contralateral pVTA microinfusion of saline (saline control). Between test days, rats received 3 days of PR training, respectively. Upon completion of behavioral testing, rats were euthanized to control for cannulae placements.

#### Histology

Histological procedures and cannula placement verification were performed as described in Experiment 1. Final sample size in Experiment 2 was *N* = 14, as two rats died during surgery, one rat was excluded because of incorrect mOFC-p cannula placement, and another animal was excluded because it failed to respond to the lever and did not receive any reward during PR testing. Moreover, two rats that were included in Experiment 1 (which targeted the mOFC-p only) were excluded in Experiment 2 due to incorrect pVTA cannulae placements. Two other rats that were not considered in Experiment 1 due to occluded guide cannulae participated in Experiment 2 (note that Experiment 2 was performed prior to Experiment 1).

### Experiment 3

Here we tested the effects of a unilateral mOFC-p microinfusion of M/B combined with either an ipsi- or contralateral pVTA microinfusion of picrotoxin in rats (initial sample size, *N* = 23) weighing 320–420 g at the onset of testing.

#### Surgery, Behavioral Procedures, and Microinfusions

For stereotaxic surgery, standard procedures were used as described in Experiment 1. Subjects received unilateral intra-mOFC-p microinfusion of M/B (125 ng each in 0.3 μL) in combination with contra- or ipsilateral intra-pVTA microinfusion of picrotoxin (100 ng/0.3 μL, Sigma-Aldrich), and all drugs were dissolved in saline. Contralateral mOFC-p/pVTA microinfusions of 0.3 μL saline served as the control.

Rats were tested for PR responding as described in Experiment 1. Each rat received three microinfusions on separate PR test days: (1) unilateral mOFC-p microinfusion of M/B combined with a contralateral pVTA microinfusions of picrotoxin, (2) unilateral mOFC-p microinfusion of M/B combined with an ipsilateral pVTA microinfusions of picrotoxin, and (3) unilateral mOFC-p microinfusion of saline combined with a contralateral pVTA microinfusion of saline. Thereafter, rats were euthanized to control for cannulae placements.

#### Histology

Cannula placements were verified as described in Experiment 1. Final sample size in Experiment 5 was *N* = 15, as one animal died during surgery. Three other animals were excluded due to an occluded guide cannula. Furthermore, two animals were excluded due to incorrect pVTA cannula placement. One animal was excluded because of incorrect mOFC-p cannula placement. Another animal was omitted because it failed to respond to the lever and did not receive any reward during PR testing.

## Results

### Experiment 1: Effects of mOFC-p Inhibition on PR Responding

**Figure 1:**
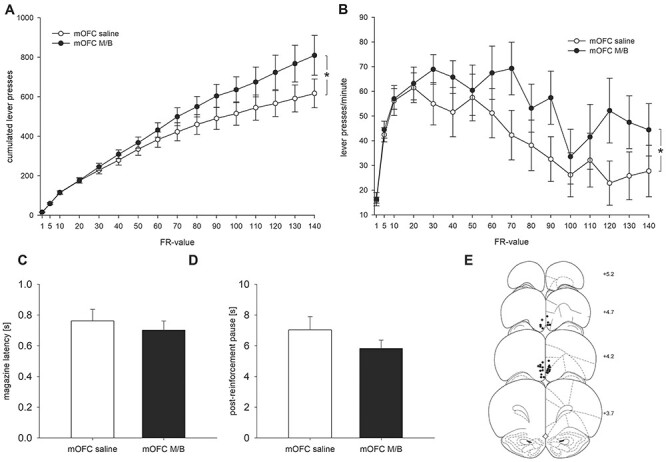
Effects of mOFC-p inhibition on PR responding. (*A*) Mean cumulative lever presses (±SEM) after intra-mOFC-p microinfusion of saline or M/B. ^*^P < 0.01: lever press × FR-value interaction, ANOVA. (*B*) Mean running lever press rates over response ratios (±SEM) after intra-mOFC-p microinfusion of saline or M/B. ^*^P < 0.01: lever press × FR-value interaction, ANOVA. (*C*) Mean magazine latencies and (*D*) postreinforcement pauses (±SEM) after intra-mOFC-p microinfusion of saline or M/B. (*E*) Schematic representation of cannula placements in animals included (*N* = 14).

Lever pressing across increasing FR-values was enhanced after intra-mOFC-p microinfusion of M/B versus saline (*N* = 14). Accordingly, an ANOVA revealed an effect of FR-value (*F*(15, 195) = 66.96; *P* < 0.01), an effect of treatment (*F*(1, 13) = 13.55; *P* < 0.01), and a FR-value × treatment interaction (*F*(15, 195) = 5.45; *P* < 0.01; Fig. 1*A*). An effect size of *r* = 0.59 was calculated for the FR-value × treatment interaction on the final FR-value, which represents a large effect. Moreover, as shown in Figure 1*B*, running lever press rates over FR-values were higher after the microinfusion of M/B versus saline. An ANOVA showed an effect of FR-value (*F*(15, 195) = 6.38; *P* < 0.01), an effect of treatment (*F*(1, 13) = 6.79; *P* < 0.05), but no effect of FR-value × treatment interaction (*F*(15, 195) = 0.94; n.s., not significant). Furthermore, magazine latencies (*t*(13) = 1.55; n.s.; Fig. 1*C*) and postreinforcement pauses (*t*(13) = 1.19; n.s.; Fig. 1*D*) did not differ. Placements of the microinfusion cannulae of all animals (Fig. 1*E*) were within the posterior subdivision of the mOFC-p (Bradfield et al. 2018). Cumulated lever presses did not differ in animals with more rostral (AP = +4.7 mm; *N* = 4) versus caudal (AP = +4.2 mm; *N* = 10) cannulae placements (data not shown). Accordingly, there was no treatment × cannulae placement interaction (*F*(1, 12) = 0.46, n.s.). An exploratory study in a small number of rats indicated that optogenetical stimulation of the mOFC-p had opposite effects and reduced PR responding (see supplementary materials).

### Experiment 2: Effects of mOFC-p/pVTA Disconnection on PR Responding

**Figure 2:**
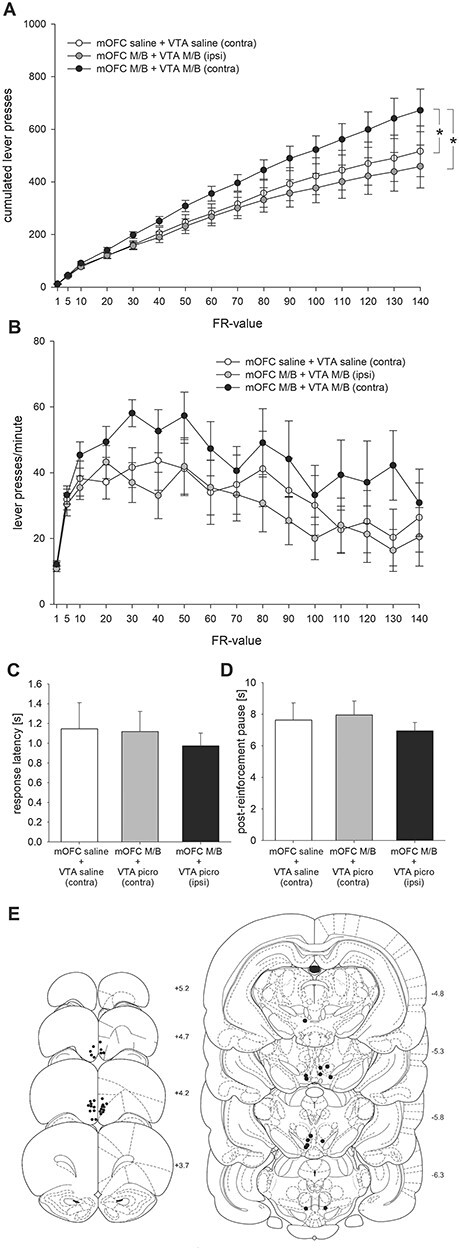
Effects of mOFC-p/pVTA disconnection on PR responding. (*A*) Mean cumulative lever presses (±SEM; ^*^P < 0.01: lever press × FR-value interaction, ANOVA); (*B*) mean running lever press rates over response ratios (±SEM); (*C*) mean magazine latencies (±SEM); and (*D*) postreinforcement pauses after unilateral mOFC-p saline + contralateral pVTA saline microinfusions, unilateral mOFC-p M/B + ipsilateral pVTA M/B microinfusions, and unilateral mOFC-p M/B + contralateral pVTA M/B microinfusions. (*E*) Schematic representation of cannula placements in animals included (*N* = 14).

Rats (*N* = 14) were subjected to unilateral mOFC-p microinfusion of M/B combined with either an ipsi- or contralateral pVTA microinfusion of M/B. Saline controls received unilateral mOFC-p and contralateral pVTA microinfusions of saline. Results show that unilateral mOFC-p/contralateral pVTA (crossed) inhibition enhanced PR responding relative to uncrossed mOFC-p/pVTA inhibition or crossed mOFC-p/pVTA saline control injections (Fig. 2*A*). An ANOVA showed an almost significant treatment effect (*F* = (2, 26) = 3.26; *P* = 0.054), an effect of FR-value (*F*(15, 195) = 54.38; *P* < 0.01) and a significant FR-value × treatment interaction (*F*(30, 390) = 2.52; *P* < 0.01). Simple effects analyses confirmed increased responding after crossed mOFC-p/pVTA inhibition compared with uncrossed mOFC-p/pVTA inhibition (*F*(15, 195) = 4.41; *P* < 0.01) or crossed mOFC-p/pVTA saline control injections (*F*(15, 195) = 2.13; *P* < 0.05). An effect size of *r* = 0.59 was calculated for FR-value × treatment interaction (crossed mOFC-p/pVTA M/B vs. crossed mOFC-p/pVTA saline) on the final FR-value, which is considered to be a large effect. Moreover, there was a trend for an effect of treatment for higher running lever press rates after crossed mOFC-p/pVTA inhibition (*F*(2, 26) = 2.94; *P* = 0.07; Fig. 2*B*), an effect of FR-value (*F*(15, 195) = 6.57; *P* < 0.01), but no effect of treatment × FR-value interaction (*F*(30, 390) = 0.58; n.s.). By contrast, magazine latencies (*F*(2, 26) = 0.20; n.s.; Fig. 2*C*) as well as postreinforcement pauses (*F*(2, 26) = 0.45; n.s.; Fig. 2*D*) did not differ.

Placements of microinfusion cannulae in the pVTA are shown in Figure 2*E*. An exploratory post hoc analysis revealed that the increase in cumulated lever presses after crossed mOFC-p/pVTA M/B versus crossed mOFC-p/pVTA saline was moderately higher in animals with more posterior cannulae placements (AP = −5.8 mm; *N* = 7) relative to more anterior cannulae placements (AP = −5.3; *N* = 7) within the pVTA (data not shown). Accordingly, we found a trend for a treatment × cannulae placement interaction (*F*(2, 24) = 2.78; *P* = 0.08).

### Experiment 3: Effects of Combined mOFC-p Inhibition/pVTA Stimulation on PR Responding

**Figure 3:**
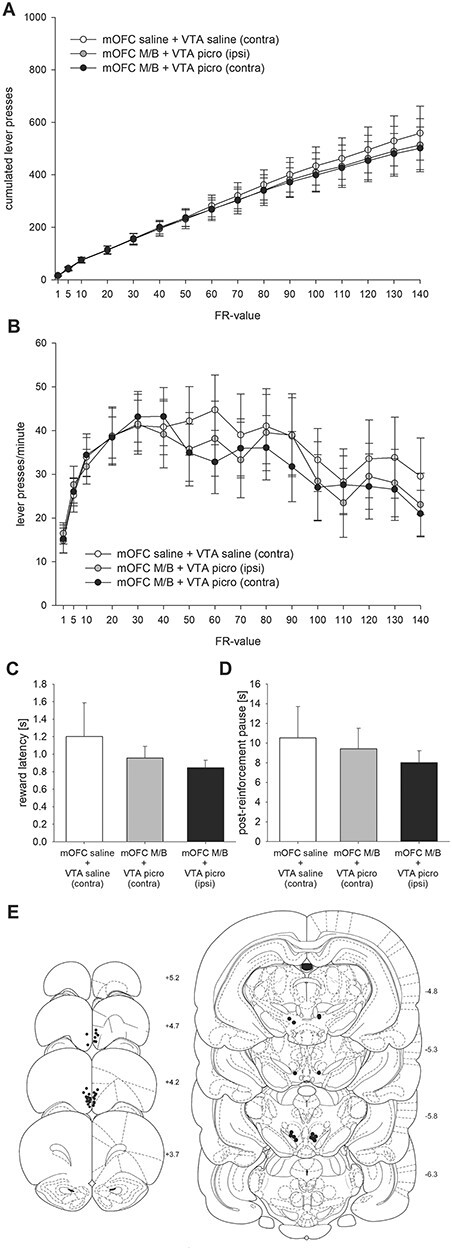
Effects of mOFC-p inhibition/pVTA stimulation on PR responding. (*A*) Mean cumulative lever presses (±SEM); (*B*) mean running lever press rates over response ratios (±SEM); (*C*) mean magazine latencies (±SEM); and (*D*) postreinforcement pauses after unilateral mOFC-p saline + contralateral pVTA saline microinfusions, unilateral mOFC-p M/B + ipsilateral pVTA picrotoxin microinfusions, and unilateral mOFC-p M/B + contralateral pVTA picrotoxin microinfusions. (*E*) Schematic representation of cannula placements in animals included (*N* = 15).

Here we tested the effects of a unilateral mOFC-p microinfusion of M/B combined with either an ipsi- or contralateral pVTA microinfusion of picrotoxin. Saline controls received unilateral mOFC-p and contralateral pVTA microinfusions of saline. Results show that crossed mOFC-p/pVTA interventions did not alter the PR responding relative to uncrossed mOFC-p/pVTA interventions or crossed mOFC-p/pVTA saline control injections (Fig. 3*A*). Also, final cumulative lever presses observed for each treatment are comparable to saline controls in Experiment 1 (see Fig. 1*A*). An ANOVA demonstrated an effect of FR-value (*F*(15, 210) = 43.50; *P* < 0.01), but no effect of treatment (*F*(2, 28) = 0.09; n.s.) and no a FR-value × treatment interaction (*F*(30, 420) = 0.21; n.s.). Moreover, running lever press rates over response ratios did not differ across treatments (Fig. 3*B*). An ANOVA showed an effect of FR-value (*F*(15, 210) = 8.18; *P* < 0.05), but no effect of treatment (*F*(2, 28) = 0.19; n.s.) and no FR-value × treatment interaction (*F*(30, 420) = 0.49; n.s.). In addition, magazine latencies (*F*(2, 28) = 0.60; n.s.; Fig. 3*C*) as well as postreinforcement pauses (*F*(2, 28) = 0.50; n.s.; Fig. 3*D*) did not differ across the treatment. Placements of microinfusion cannulae are shown in Figure 3*E* (*N* = 15).

## Discussion

Here we show that the mOFC-p controls effort-related responding through interactions with pVTA GABAergic mechanisms. These findings provide novel evidence for a key role of the mOFC-p and pVTA in mediating the willingness to expend effort to reach a goal.

### The mOFC-p Supports PR Responding

In previous studies, the involvement of the mOFC in effort-related responding has been examined in PR tasks using a breakpoint, which is defined as the ratio at which an animal stops responding (Gourley et al. 2016; Münster and Hauber 2018). However, the specificity of the breakpoint as a motivational index has been questioned (Stewart 1975; Bradshaw and Killeen 2012). For instance, it is not an unambiguous criterion that an animal indeed stopped responding. Furthermore, it is derived from a single within-session time point, while the data from the rest of the session are being ignored. To circumvent these issues, in the current PR task variant, we implemented a schedule of ratio increases that demanded increasingly higher response rates but prevented animals to stop lever pressing. Consistent with this notion, cumulated lever presses became constantly higher, even with the final FR-values. Also, response rates declined in a curvilinear fashion but did not reach zero. Relative to the PR task version used in our earlier study, we also constricted session time to 16 min (as opposed to 60 min) to lower possible confounding effects of increasing within-session motor fatigue and satiety. In addition, reduction of session time makes this task more appropriate for optogenetic interventions, for instance, because time-dependent nonspecific light effects such as local heating of tissue (Yizhar et al. 2011) are markedly reduced. Accordingly, our Supplementary data indicate that the PR task is sensitive to the behavioral effects of optogenetic manipulation.

Consistent with our previous study (Münster and Hauber 2018), pharmacological inhibition of the mOFC-p markedly increased cumulative lever presses relative to controls. Notably, effect sizes are almost identical (*r* = 0.59 and *r* = 0.62 in Experiment 1 and in Münster and Hauber 2018, respectively), a finding that indicates that the current PR task modifications, if even, only marginally influenced task sensitivity. Yet, the observation that mOFC-p inactivation equally enhanced responding in two PR task variants that entail markedly different PR schedules provides strong support to the notion that mOFC-p control of effort-related function is fundamental and not particularly sensitive to the specific response costs imposed by the task. Results further show that, relative to vehicle controls, mOFC-p inhibition moderately increased peak running response rates and reduced the decay of running response rates. Theoretical models and empirical accounts suggest that, in PR tasks, treatments that promote effort-related function are associated with higher peak response rates, while treatments that enhance reinforcer efficacy, for example, increases of reward magnitude, selectively reduce the rate of decay of response rates, with higher reinforcer efficacy being associated with a more gradual decline (Bradshaw and Killeen 2012). Our data provide evidence for a combination of these effects, suggesting that increased PR responding after mOFC-p inhibition could reflect both an overestimation of reward magnitude and a facilitation of high-effort responding. The mOFC-p could thus play a role in assessing response costs and benefits to set the threshold for response costs that will be tolerated to obtain a reward. Consistent with this notion, neural activity in the OFC encodes reward magnitude and costs in terms of time or effort required to receive a reward (Rudebeck and Rich 2018). Also, a recent study using an effort-based cost–benefit task in humans reveals that mOFC-p signals represent a composite value of effort level and reward type (Park et al. 2017). Moreover, our Supplementary experiments suggest that photostimulation of the mOFC-p can reduce cumulative lever pressing. In line with this observation, pharmacological stimulation of the mOFC-p (Münster and Hauber 2018) or activation of stimulatory G_s_-DREADDs in the mOFC decreased PR responding (Gourley et al. 2016). Together, these data are consistent with earlier findings demonstrating a bidirectional control of effort-related function by the mOFC-p (Münster and Hauber 2018). Higher or lower mOFC-p neural activity appears to be permissive of lower or higher cost expenditure to obtain a reward.

Novel evidence suggests that the mOFC, in terms of connectivity and behavioral function, differs along its anterio-posterior axis. For instance, anatomical studies revealed that the anterior mOFC has greater connectivity with the nucleus accumbens core than the mOFC-p (Bradfield et al. 2018). In functional terms, the anterior mOFC specifically supports the retrieval of value memory for the use in guiding actions and enables predictions about action outcomes, while its role in effort-related function is yet unexplored (Izquierdo 2017; Bradfield et al. 2018; Woon et al. 2019; Bradfield and Hart 2020). By contrast, the mOFC-p does not govern value memory retrieval, but—as shown here and in earlier studies—controls effort-related function (Bradfield et al. 2018; Münster and Hauber 2018).

### The mOFC-p Supports PR Responding Through Interactions with the pVTA

Relative to mOFC-p/pVTA control interventions, disconnecting the mOFC-p from the pVTA by asymmetric infusion of GABA_A/B_ receptor agonists enhanced cumulative lever presses, a treatment effect that was associated with a large effect size. Moreover, overall running lever press rates tended to be increased in animals subjected to a disconnection and, relative to controls, show a higher peak rate and a delayed decrease of response rates across higher ratios. This pattern of effects induced by the mOFC-p/pVTA disconnection is consistent with the one induced by bilateral pharmacological mOFC-p inactivation and suggests that the mOFC-p governs PR responding via inhibition of GABAergic mechanisms in the pVTA. Remarkably, a unilateral M/B infusion into the mOFC-p in combination with a contralateral infusion of picrotoxin into the pVTA had no or even marginally inhibitory effects on lever pressing, indicating that a stimulation of GABAergic mechanisms in the pVTA may not increase PR responding. Accordingly, microinjections of muscimol into the pVTA stimulated motor activity (Arnt and Scheel-Krueger 1979; Wirtshafter and Klitenick 1989), while microinjections of picrotoxin had no motor effects (Arnt and Scheel-Krueger 1979). Because muscimol and picrotoxin microinfusion into the anterior VTA had inverse effects on motor activity (Arnt and Scheel-Krueger 1979), our findings imply regionally selective drug actions within the pVTA. Notwithstanding, to analyze the topographical and regional organizations of the VTA in more detail, comparative experiments analyzing, for example, the effects of disconnection of mOFC-p and anterior versus posterior VTA on PR responding and VTA c-Fos expression are warranted.

Collectively, our data demonstrate that the mOFC-p mediates effort-related motivational function through interactions with GABAergic mechanisms in the pVTA. It is conceivable that increased PR responding after mOFC-p inhibition could be accounted for by a reduced mOFC-p excitatory drive onto the pVTA GABA interneurons.

### mOFC-p/pVTA Interactions in Effort-Related Motivation: Functional Implications

In view of the intricate cellular and structural heterogeneity of the VTA as well as its multifaceted neuronal connectivity (Morales and Margolis 2017), behavioral effects of pVTA GABAergic manipulation seen here likely involve a variety of neural and neurochemical mechanisms. Because of their key role in PR responding, VTA dopamine neurons are one major target of the mOFC-p to support effort-related motivational function (Salamone and Correa 2012; Boekhoudt et al. 2018). Given the antagonistic GABA/DA interactions in the pVTA (Kalivas et al. 1990; Kalivas 1993; Boehm 2nd et al. 2002), it seems plausible that a unilateral stimulation of mOFC-p GABAergic transmission in combination with a contralateral stimulation of pVTA GABAergic transmission enhanced dopamine neuron activity in both hemispheres thereby invigorating PR responding. While our data provide no direct support to this notion, considerable evidence suggests that prefrontal neurons control VTA dopamine neuron activity via VTA GABAergic mechanisms. For instance, electrical stimulation of the OFC inhibited VTA dopamine neurons, probably involving a GABAergic relay (Takahashi et al. 2011). In turn, prefrontal microinjection of a GABA_A_ agonist elevated dopamine neuron responses to reward-predicting stimuli (Jo et al. 2013). The authors concluded that a reduced prefrontal input promotes dopamine neuron responses to reward-predicting stimuli via VTA GABA interneurons. Of note, we recently found that a blockade of dopamine D1 receptors in the mOFC-p reduced PR responding (Münster et al. 2020). Therefore, we cannot exclude that disconnection of mOFC-p and pVTA increased the activity of dopamine neurons projecting to the mOFC-p, an effect that may contribute to enhanced PR responding. However, this mechanism alone may not account for the observed effects. It is well known that VTA dopamine neurons project to the nucleus accumbens and the prefrontal cortex, yet, prefrontal regions receive much less dopaminergic innervation from the VTA than does the nucleus accumbens (Ikemoto 2007).

Rodent studies of effort-related processes not only can provide insights into the neural circuitry and neurochemistry of motivation but could also provide a further understanding of the neural basis of effort-related dysfunction in human psychopathologies (Salamone and Correa 2012). Clinical studies have demonstrated altered effort-based responding in psychiatric disorders associated with OFC dysfunction, such as depression (Price and Drevets 2010; Treadway et al. 2012; Yang et al. 2014, 2016; Culbreth et al. 2018) and schizophrenia (Gold et al. 2013; Liu et al. 2014; Culbreth et al. 2017). For instance, in a “Effort Expenditure for Rewards Task,” patients with depression were less willing to expend effort for rewards than controls and were less able to use information about the magnitude of rewards to guide behavior (Treadway et al. 2012). Our results in rodents point to the possibility that effort-related motivational dysfunction in these psychopathologies involves altered mOFC-p/pVTA interaction.

## Supplementary Material

FigS1_tgaa086

FigS2_tgaa086

FigS3_tgaa086

OFC_Paper_Final_Supplement_tgaa086

## Notes

We thank I. Newar for expert help in animal husbandry and M. Münkel for providing assistance with Supplementary Figure S2. We acknowledge the UNC Gene Therapy Vector Core and Dr R. Jude Samulski. *Conflict of Interest*: None declared.
