# Supplementary figures and images for: Role of the Medial Orbitofrontal Cortex and Ventral Tegmental Area in Effort-Related Responding
Source: Cereb Cortex Commun. 2020 Nov 26;1(1):tgaa086. doi: 10.1093/texcom/tgaa086 (PMC8152852; doi:10.1093/texcom/tgaa086)

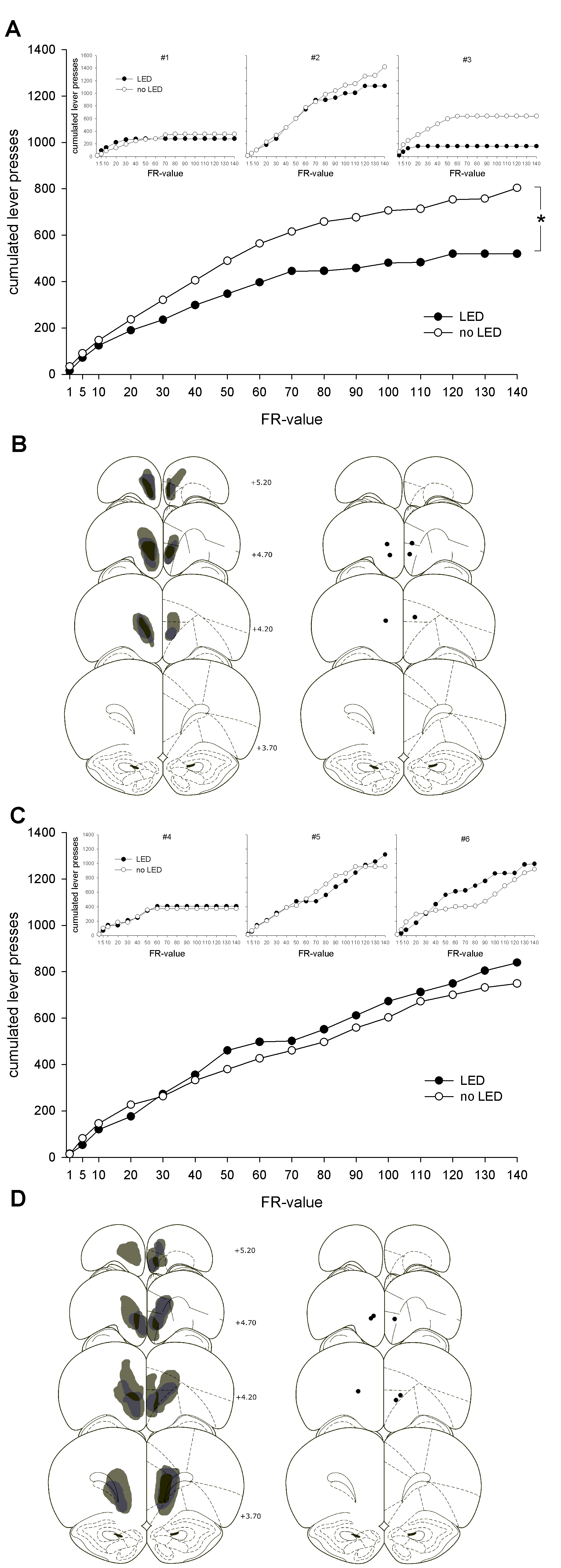

Supplement: FigS1_tgaa086 [file figs1_tgaa086.gif]

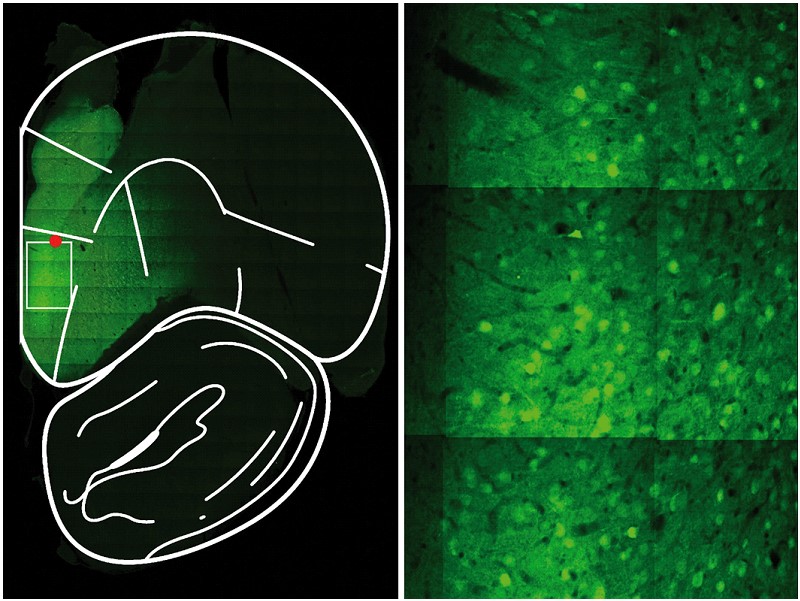

Supplement: FigS2_tgaa086 [file figs2_tgaa086.jpeg]

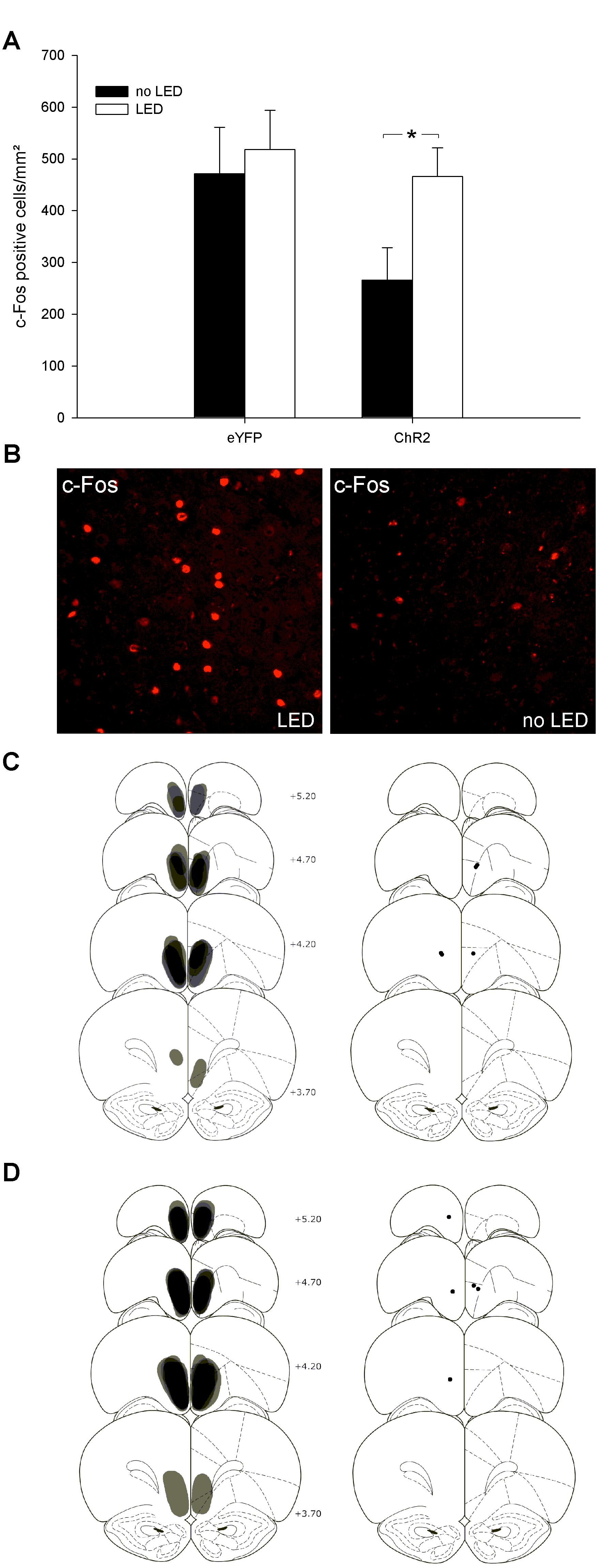

Supplement: FigS3_tgaa086 [file figs3_tgaa086.gif]
