# Supplementary material for: Role of the Medial Orbitofrontal Cortex and Ventral Tegmental Area in Effort-Related Responding
Source: Cereb Cortex Commun. 2020 Nov 26;1(1):tgaa086. doi: 10.1093/texcom/tgaa086 (PMC8152852; doi:10.1093/texcom/tgaa086)
Supplement: OFC_Paper_Final_Supplement_tgaa086 [file ofc_paper_final_supplement_tgaa086.docx]

**Role of the Medial Orbitofrontal Cortex and Ventral Tegmental Area in Effort-Related Responding**

**Supplemental Information**

In two supplemental experiments, we explored the effects of optogenetic mOFC-p stimulation on PR responding (Experiment S1) and c-Fos expression in the mOFC-p (Experiment S2).

**Experiment S1: Supplemental Materials and Methods**

*Subjects and behavioral procedures*

Rats (initial sample size N=8) weighing 370g - 480g at the onset of testing received bilateral intra-mOFC-p microinjections of rAAV5/CamkII-hChR2(H134R)-EYFP-WPRE-PA (“ChR2 rats”) or rAAV5/CamkIIa-eYFP (“eYFP rats”). Thereafter, ChR2 and eYFP rats were subjected to one magazine training session (30 min) in which casein pellets (45-mg dustless precision pellets®; Bioserv, Frenchtown, NJ, USA) were delivered on an independent random-time schedule (RT-60) with the lever withdrawn as described for Experiment 1. After magazine training, rats were given 2 sessions on a CRF schedule where each lever press resulted in the delivery of one pellet. For unknown reasons, basal lever press rates in this cohort of rats were lower than in other experiments. To enhance lever press performance, animals were therefore shifted to a random ratio schedule (RR) of reinforcement before being trained in the PR task (day 3-4 = RR-5, P (O|A) = 0.2; day 5–6 = RR-10, P (O|A) = 0.1; day 7-11 = RR-20, P (O|A) = 0.05). Thereafter, animals were trained in the PR task until they showed robust baseline responding, i.e. stable total lever presses across session, and were then implanted with optic fiber stubs. After recovery, ChR2 and eYFP rats were tested in the photostimulation (“LED”) *vs.* no-photostimulation (“no LED”) condition using a within-subject design on 2 separate testing days. Thereafter, rats were euthanized to control for fiber stub placements. eYFP expression was assessed using a ZEISS Axio Observer Z1 microscope under a 20x objective. Images were taken using an Axiocam 503.

*Surgery*

For stereotaxic surgery, rats were anesthetized with a mixture of ketamine (90mg/kg i.p.; Medistar GmbH, Ascheberg, Germany) and xylazine (10mg/kg Rompun i.p.; Bayer AG, Leverkusen, Germany) and secured in a stereotaxic apparatus with atraumatic ear bars (David Kopf Instruments). Standard stereotactic procedures were used. Rats received either 0.4µL of the adeno-associated virus rAAV5/CamkII-hChR2(H134R)-EYFP-WPRE-PA (UNC Gene Therapy Vector Core, Thurston Bowles, Chapel Hill, NC, USA; N=3) or of the adeno-associated control virus rAAV5/CamkIIa-EYFP (UNC Gene Therapy Vector Core, Thurston Bowles, Chapel Hill, NC, USA; N=5) bilaterally into the mOFC-p. Coordinates with reference to Paxinos and Watson (1997) were as follows: AP +4.5mm; ML ± 0.6mm; DV -4.3mm. Each microinjection was made over 4 min at an infusion rate of 0.1µL⁄min, the needle was left in place for an additional 5 min to allow for diffusion. Postoperative recovery was at least 7 days before behavioral training.

After showing a robust baseline in the PR task, rats were implanted with guide cannulae. Subjects were anesthetized with a mixture of ketamine (90mg/kg i.p.; Medistar GmbH, Ascheberg, Germany) and xylazine (10mg/kg Rompun i.p.; Bayer AG, Leverkusen, Germany) and secured in a stereotaxic apparatus with atraumatic ear bars (David Kopf Instruments). Optic fiber stubs (200 µm core diameter, Standard PlexBright Fiber Stub Implants, Plexon, USA) were implanted bilaterally using standard stereotactic procedures. They were positioned ~0.5mm dorsal to the mOFC-p using the following coordinates with reference to (2): AP +4.5 mm; ML ±1.7 mm; DV -3.7 mm at an angle of 15°. Postoperative recovery was at least 7 days before behavioral training and testing.

*Optogenetic manipulation*

Bilateral photostimulation stimulation of the mOFC-p was performed using PlexBright Compact LEDs (300mA, 465nm) and PlexBright 200/230µm high durability 0.5NA fibers. ChR2 and eYFP rats were tested in the photostimulation (“LED”) *vs.* no-stimulation (“no LED”) condition using a within subject design on two separate testing days. In the photostimulation condition, optical stimulation was given for 30 min immediately prior to test onset at a rate of 20Hz for 10s/min (10ms pulse duration, 200 pulses/min). The photostimulation protocol used here has been employed in previous studies and shown to interfere with PR responding and neuronal activity *in vitro* (Larson et al., 2015). Furthermore, the photostimulation pattern used here has been shown to depolarize ChR2-expressing prefrontal pyramidal neurons *in vitro*, and increased c-Fos expression in ChR2-expressing neurons (Villaruel et al., 2018). Light intensity was adjusted to ~15mW intensity at each tip. In the no stimulation condition, rats were tethered to the fibers without LED light stimulation for 30 min immediately prior to test onset.

*Histology*

After completion of behavioral testing, rats were euthanized and perfused transcardially with 0.01% heparin sodium salt in PBS, followed by 4% paraformaldehyde in PBS. Brains were removed, postfixed in paraformaldehyde for 24 h. Thereafter, brains were stored in PBS containing 0.1% sodium azide (Carl Roth GmbH + Co. KG, Karlsruhe, Germany) until slicing. Coronal sections (55 µm) were made with a vibratome (HM 650V Vibrating Blade Microtome; Thermo Fisher Scientific, Karlsruhe, Germany). Sections were blocked in a PBS-T blocking solution containing 10% Normal Horse serum (NHS, S-2000), Vector Laboratories, Inc., 30 Ingold Road, Burlingame, CA USA) and 0.3% Triton for 90 min. Thereafter, sections were incubated overnight at room temperature in a primary antibody PBS solution containing GFP: goat anti -GFP (1:75; GeneTEX, Inc., USA), 2% NHS and 0.06% Triton. Sections were washed three times for 10 min each with PBS before being incubated at room temperature for 3h in a PBS solution containing 2% NHS, 0.06% Triton and the secondary antibody donkey anti -goat 488 (1:1000). Sections were washed 3 times for 10 min in PBS before being stained with NeuroTrace 435/455 Blue Fluorescent Nissl Stain (1:500; N21479; Invitrogen™; Thermofisher, USA). Thereafter, sections were washed again 3 times in PBS for 10 min each and mounted on slides before being coverslipped with Antifade Mounting Medium (VECTASHIELD® Vibrance™, Burlingame, CA 94010, USA) and coverslips.

Two animals were excluded in the eYFP group because of incorrect fiber stub placement. Localization of eYFP expression and fiber stub placements of rats included in the experiment are shown in Fig. S1D (ChR2, N=3) and Fig.S1H (eYFP, N=3).

**Experiment S2: Supplemental Materials and Methods**

*Subjects and procedures*

Rats (initial sample size N=16) weighing 360g - 490g (23-28 weeks) at the onset of testing were used in this experiment. ChR2 and eYFP rats received unilateral photostimulation of the mOFC-p for 30min. eYFP and c-Fos expression were assessed using a ZEISS Axio Observer Z1 microscope under a 20x objective. Expression of c-Fos in the mOFC-p was assessed in the photostimulated mOFC-p and compared with c-Fos expression in the not-stimulated mOFC-p in the other hemisphere (within-subject comparisons). Two images per hemisphere within the mOFC-p were taken using an Axiocam 503. Neurons expressing c-Fos were counted by an observer blind to the experimental conditions. For each animal, the mean number of c-Fos positive cells in both images was calculated and scaled to c-Fos positive cells/mm².

*Surgery and photostimulation*

Fiber stubs were implanted unilaterally using procedures described in Experiment S1. Habituation training started 3 weeks post-surgery, testing 4 weeks post-surgery. For optogenetic stimulation, the protocol of Experiment S1 was used.

*Histology*

Two hours after stimulation onset, rats were euthanized and perfused transcardially with 0.01% heparin sodium salt in PBS, followed by 4% paraformaldehyde in PBS. The brains were removed, postfixed in paraformaldehyde for 24 h. Thereafter, brains were stored in PBS containing 0.1% sodium azide (Carl Roth GmbH + Co. KG, Karlsruhe, Germany) until slicing. Coronal sections (35 µm) were made. Sections were blocked in a PBS-T blocking solution containing 10% Normal Horse serum (NHS, S-2000, Vector Laboratories, Inc.,30 Ingold Road, Burlingame, CA USA) and 0.5% Triton for 90 min. Thereafter, sections were incubated for 36h at room temperature in the primary antibody PBS solution containing c-Fos monoclonal antibody (1:3000; MA5-15055; Invitrogen™, Thermo Fisher Scientific, Karlsruhe, Germany), goat anti-GFP (1:750; GTX 26673, GeneTEX, Inc., USA), 2% NHS and 0.2% Triton. Sections were washed three times for 10 min each with PBS before being incubated at room temperature for 90min in a PBS solution containing 2% NHS, 0.2% Triton and the secondary antibodies donkey anti-goat IgG (1:1000; Alexa Fluor 488; A-11055; Invitrogen™, Thermo Fisher Scientific, Karlsruhe, Germany) and donkey anti-rabbit IgG (1:1000; Alexa Fluor 555; A-31572; Invitrogen™; Thermo Fisher Scientific, Karlsruhe, Germany). Sections were washed 3 times for 10 min in PBS. Thereafter, sections were mounted on slides and coverslipped with antifade mounting medium (VECTASHIELD® Vibrance™, Burlingame, CA 94010, USA) and coverslips.

Three animals died during surgery (2 of the ChR2 group, 1 of the eYFP group). Another animal of the ChR2 group was excluded because there was no eYFP expression. In the eYFP group one animal was excluded because there were no eYFP positive cell bodies. Furthermore, one animal was excluded due to fiber stub misplacement. Thus, the final sample sizes were N=5, respectively.

**Experiment S1: Supplemental Results**

**Effects of optogenetic mOFC-p stimulation on PR responding**

Relative to pharmacological manipulation, ChR2 and eYFP rats displayed after optogenetic manipulation higher inter-individual variability, which could be accounted for by within-task tethering with optofibers. Photostimulation of the mOFC-p in ChR2 rats (N=3) prior to test onset reduced lever pressing across increasing FR-values relative to the non-stimulation condition. An ANOVA revealed an effect of FR-value (F(15,30) = 3.29; p<0.01), an interaction of FR-value x stimulation condition (F(15,30)=3.96; p<0.01), but no effect of stimulation condition (F(1,2)=1.99; ns.) (Fig. S1A). An effect size of r=0.93 was calculated for the FR-value x treatment interaction on the final FR-value which represents a large effect. By contrast, magazine latencies (t(2)=1.30; ns.) and post-reinforcement pauses (t(2)=0.53; ns.) did not differ across stimulation conditions (include means/SEM). Photostimulation in eYFP controls (N=3) did not alter lever pressing relative to the non-stimulation condition (Fig. S1C). An ANOVA revealed an effect of FR-value (F(15,30)=12.02; p<0.01), but no effect of stimulation condition (F(1,2)=0.82; ns.) and no FR-value x stimulation condition interaction (F(15,30)=0.50; ns.). In addition, magazine latencies (t(2)=5.71; p<0.05), but not post-reinforcement pauses (t(2)=1.53; ns.) differed between stimulation conditions (include means/SEM). Schematic representations of eYFP expression and fiber stub placement of all rats are shown in Fig. S1B (ChR2 rats) and Fig. S1D (eYFP rats). Also, microphotographs showing eYFP expression and fiber stub placement in an individual ChR2 rat are given in Fig. S2.

Taken together, this experiment provides initial evidence that photostimulation of the mOFC in ChR2 rats prior to test onset can reduce within-session cumulative lever pressing relative to the no-photostimulation condition. Control experiments comparing photostimulation *vs.* no-photostimulation in eYFP rats suggest that light delivery *per se* had no nonspecific effects on PR responding. Moreover, as PR responding under no-photostimulation conditions was similar in ChR2 and eYFP rats, ChR2 expression as such may be devoid of nonspecific behavioral effects. In ongoing studies, we try to substantiate these findings by testing within-session mOFC optogenetic manipulations at discrete time points

**Experiment S2: Supplemental Results**

**Effects of optogenetic mOFC-p stimulation on c-Fos expression**

Photostimulation in the mOFC-p of ChR2 rats (N=5) increased mOFC-p c-Fos expression relative to the non-stimulation condition (“no LED”: 266.23±61.96 c-Fos positive cells/mm²; “LED”: 466.34±55.10 c-Fos positive cells/mm²; Z=2.02; p<0.05) (Fig. S3A). Microphotographs showing c-Fos expression in an individual ChR2 rat in both stimulation conditions are shown in Fig. S3B. In eYFP rats (N=5), mOFC-p photostimulation did not alter c-Fos expression in the mOFC-p relative to the non-stimulation condition (“no LED”: 471.49±89.74 c-Fos positive cells/mm²; “LED”: 517.87±75.69 c-Fos positive cells/mm²; Z=0.13; ns.). The eYFP expression and fiber stub placement in an individual ChR2 and eYFP rat as well as a schematic representation of the eYFP expression and fiber stub placement in all ChR2 and eYFP rats are shown in Figs. S3C and S3D.

Overall, our data indicate that photostimulation increased neuronal activation in the mOFC of ChR2 rats. Likewise, Larson et al. (2015) demonstrated that photostimulation according to the protocol employed here interfered with neuronal activity *in vitro* and PR responding. Control experiments in eYFP rats comparing photostimulation *vs.* no-photostimulation indicate the absence of nonspecific effects of light delivery. The finding that expression of c-Fos was higher in non-stimulated eYFP *vs.* CR2 rats may be accounted for by testing eYFP and ChR2 rats on separate days. c-Fos expression is known to be highly sensitive to testing conditions such as forms of handling or time-of-day (Asanuma and Ogawa, 1994; Krukow, 1999). However, to limit the influence of variable testing conditions, we used a within-subject design comparing photostimulation *vs*. no-photostimulation in the same individuals.

**Supplemental References:**

Asanuma M, Ogawa, N. 1994. Pitfalls in Assessment of c-fos mRNA Expression in the Brain:

Effects of Animal Handling. Rev Neurosci 5:171-178

Krukow, TL. 1999. c-fos Expression as a Marker of Functional Activity in the Brain. In Neuromethods, Vol. 33 Cell Neurobiology Techniues p.213-230. Eds. Boulton A, Baker GB, Bateson AN; Humana Press.

Larson EB, Wissman AM, Loriaux AL, Kourrich S, Self DW. 2015. Optogenetic stimulation of accumbens shell or shell projections to lateral hypothalamus produce differential effects on the motivation for cocaine. J Neurosci 35:3537-3543.

Villaruel FR, Lacroix F, Sanio C, Sparks DW, Chapman CA, Chaudhri N. 2018. Optogenetic Activation of the Infralimbic Cortex Suppresses the Return of Appetitive Pavlovian-Conditioned Responding Following Extinction. Cereb Cortex 28:4210-4221.

**Supplemental Figure Legends**

**Fig. S1.** Effects of mOFC-p photostimulation on PR responding. **(A)** Individual and mean cumulative lever presses (±SEM) after after photostimulation (“LED”) or non-stimulation (“no LED”) of the mOFC-p in ChR2 rats (N=3). *p<0.01: lever press x FR-value interaction, ANOVA. **(B)** Schematic representations of eYFP expression and fiber stub placement in ChR2 rats **(C)** Individual and mean cumulative lever presses (±SEM) after photostimulation (“LED”) or non-stimulation (“no LED”) of the mOFC-p in eYFP rats (N=3). **(D)** Schematic representations of eYFP expression and fiber stub placement in eYFP rats.

**Fig. S2.** Microphotographs showing the eYFP expression and fiber stub placement in the mOFC-p in a ChR2 rat. Right panel: Boxed area in higher magnification.

**Fig. S3.** Effects of mOFC-p photostimulation on c-Fos expression. **(A)** Microphotographs showing mOFC-p c-Fos expression in a ChR2 rat with (“LED”) and without (“noLED”) photostimulation. Schematic representations of the eYFP expression and fiber stub placement in all **(B)** ChR2 (N=5) and **(C)** eYFP rats (N=5).
